# Successful Treatment with Clonazepam and Pramipexole of a Patient with Sleep-Related Eating Disorder Associated with Restless Legs Syndrome: A Case Report

**DOI:** 10.1155/2012/893681

**Published:** 2012-03-14

**Authors:** Nobuyuki Kobayashi, Ryohei Yoshimura, Masahiro Takano

**Affiliations:** ^1^Department of Psychosomatic Medicine, Takano Hospital, 4-2-88 Obiyama, Kumamoto 862-0924, Japan; ^2^Oyama Medical Clinic, 6-10-15 Oyama, Kumamoto 861-8045, Japan; ^3^Coloproctology Center, Takano Hospital, 4-2-88 Obiyama, Kumamoto 862-0924, Japan

## Abstract

Sleep-related eating disorder (SRED) is characterized by recurrent episodes of involuntary eating during sleep period and is often associated with restless legs syndrome (RLS). Although pharmacotherapy is recommended for SRED patients, no drug have shown promising effects so far. The patient, a 48-year-old Japanese housewife, first visited our clinic and complained about nighttime eating. She had a history of hypertension, diabetes mellitus, sleep apnea syndrome, and depression. Insomnia appeared 10 years before the first visit and she often received hypnosedatives; at the same time, she developed nocturnal eating episodes. She had amnesia for these episodes, and she felt urge to move her legs while sleeping. The patient was diagnosed with SRED and RLS. Reduction in the doses of triazolam decreased her nighttime eating frequency, and her complete amnesia changed to vague recall of eating during night. Clonazepam 1.0 mg at bedtime decreased nocturnal eating frequency from 1 to 2 times per month, though sleepwalking remained. Administration of pramipexole 0.125 mg relieved all symptoms including SRED, RLS, and sleepwalking. This is the first paper to report that the combination of clonazepam and pramipexole therapy-reduced SRED episodes and RLS symptoms.

## 1. Introduction

Sleep-related eating disorder (SRED) is characterized by recurrent episodes of involuntary eating and drinking during the main sleep period. Several patients have amnesia for the events, and they eat during night usually without hunger or thirst and at different consciousness levels. The patients often eat unpalatable substances such as frozen foods and cigarettes [1]. Although the prevalence of this condition is high, nearly 5% in the general population [2], SRED is an underrecognized condition by majority of clinicians.

SRED is thought to be a sleep disorder and is distinct from nocturnal eating disorder (NES) [3]. Patients with NES exhibit nocturnal hyperphagia, insomnia, and morning anorexia. They are aware of nocturnal arousal and hyperphagia.

SRED is often associated with restless legs syndrome (RLS) [4]. RLS is a neurological disorder characterized by an irresistible urge to move the legs, especially at rest. The symptoms worsen in the evening and night and improve with activity such as walking. Besides RLS, SRED is often associated with other sleep-related disorders including periodic limb movements of sleep (PLMS), somnambulism, parasomnia such as sleepwalking, and sleep apnea syndrome (SAS) [4].

Pharmacotherapy is recommended for SRED patients [5]. Although several drugs have been reported to be useful for SRED patients [5–8], a standard drug has not yet been found. Pramipexole is shown to be effective in about 75% of RLS patients [9]. In the patients with SRED, pramipexole decreased nocturnal motor activity and improved sleep quality in a small trial, but it failed to lower the frequency of nocturnal eating [6].

We report the case of a patient with SRED and RSL who received combined treatment of clonazepam and pramipexole that reduced the frequency of night eating as well as the urge to move legs during sleeping.

## 2. Case Presentation

 The patient, a 48-year-old Japanese housewife, first visited the psychosomatic clinic and complained of nighttime eating. She had history of hypertension, type 2 diabetes mellitus (T2DM), and depression. Insomnia appeared 10 years before the first visit, and she often received hypnosedatives. At the same time, nocturnal eating episodes developed which occurred at about half of nights. She ate without hunger and the eating episodes were often repeated several times in a night. Sometimes she consumed substances other than foods such as a soap cake and cooked or ordered foods through the internet. She had complete amnesia or partial recall of these episodes and was surprised to find the remains of night eating on the next morning. Five years after the start of these episodes, the frequency of night eating increased. Further, her blood glucose level increased without any change in weight and her snoring increased. She felt the urge to move her legs while sleeping, which was decreased by physical movement.

 Two years before the first visit, she was diagnosed with sleep apnea syndrome (SAS) and her apnea hypopnea index was 34.9 (normal range, <5 events/h) as shown by pulse oximeter. Treatment with continuous positive airway pressure was recommended, but she hoped to follow up.

At the first visit, her physical examination data was as follows: height, 155 cm; body weight, 55 kg; blood pressure, 128/78 mmHg. The ophthalmologic and neurological examination findings were normal. Urinalysis revealed no proteinuria, and complete blood cell count was within the normal limit. Serum chemistry revealed no abnormalities except high fasting blood glucose level (147 mg/dL; normal, <110 mg/dL) and high-hemoglobin A1c (HbA1c) level (7.0%: normal range, 4.4–5.8%). Her hormone profile was as follows: serum thyroid-stimulating hormone (TSH) level, 2.0 *μ*IU/mL (range: 0.4–4.0 *μ*IU/mL); free thyroxine (fT4) level, 1.0 ng/dL (range: 0.8–1.9 ng/dL); cortisol level, 18.7 *μ*g/dL (range: 3.9–20.6 ng/dL). 

Psychometric data were as follows. The self-rated depression scale (SDS) score was 49 (normal, <48). Minnesota multiphasic personality inventory showed neurotic tendency, and the subscales were as follows (only *T* scores ≥70): hypochondriasis, 78.9; depression, 76.6; hysteria, 73.7. The score on International Restless Legs Syndrome rating scale (IRLS) was 31 points (very severe).

**Figure 1:**
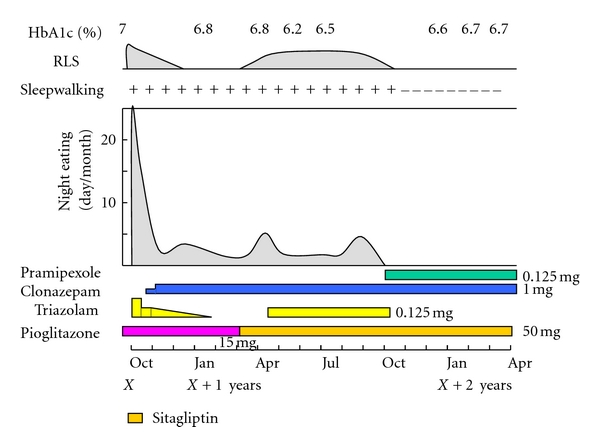
Clinical course. Reduction in the dose of triazolam decreased the freque006Ecy of night eating. Night eating was reduced by stepwise addition of clonazepam and pramipexole. RLS: restless legs syndrome; Sleepwalking +: present; Sleepwalking −: absent; *X* year presents the year at first visit.

She was diagnosed with SRED and RLS, and was advised to lower the dosage of triazolam from 0.25 to 0.125 mg. The night eating frequency decreased from almost every night to about a half of the nights, and complete amnesia changed to vague recall of the night eating (Figure 1).

Then, we included clonazepam from 0.5 to 1.0 mg at bedtime, which decreased the frequency of nocturnal eating 1 to 2 times per month. However, she continued sleepwalking; she would stand up or walk about for a few minutes when aroused. Her urge to move legs was reduced after discontinuing triazolam.

 In a few months, nocturnal eating frequency increased up to 5 nights per month and the urge to move legs and parasomnia also increased. Since she was engaged in caring for her family members who were undergoing treatment for some diseases at that time, psychotherapy treatment was administered to resolve her cognitive behavioral problems. Clonazepam up to 1.5 mg failed to achieve additional improvement, and subsequently, pramipexole 0.125 mg was started. Sleepwalking disappeared within two days and night eating episodes, and the urge to move legs also reduced after starting pramipexole. The IRLS score was lowered to 13 points (moderate). She has been maintaining this condition for 10 months and at presentation.

Slight improvement of plasma HbA1c level was observed immediately after reduction in the night eating episodes. No apparent improvement was found after changing the drug for T2DM from pioglitazone to sitagliptin. She experienced no adverse effect.

## 3. Discussion

 In our patient, SRED was improved with 3 steps involving reduction in the dose of triazolam and subsequent medication with clonazepam and pramipexole. Many psychotropic drugs were reported to induce or promote SRED, including triazolam, amitriptyline, olanzapine, and zolpidem [5, 10–13]. Discontinuation of triazolam was abandoned because she felt anxious to stop hypnosedatives.

Clonazepam is effective in some patients with RLS and is used for treating patients with SRED-associated sleep disorders [5]. Although clonazepam is useful for reducing the frequency of nocturnal eating with a low response rate, a combination of dopaminergic agents and clonazepam was shown to be effective for SRED-associated sleep disorders with relative high response rate [5]. Clonazepam monotherapy remarkably decreased the frequency of nocturnal eating in our patient.

 Pramipexole is a dopaminergic agent effective for RLS and Parkinson's disease [9], and it has been recently made available in Japan. Frequent coexistence of SRED and RLS suggested some relationship between SRED and RSL [14], and pramipexole was expected to be useful for SRED as well as RLS. This hypothesis has been previously tested. The results of a small double-blind, placebo-controlled trial using pramipexole in patients with SRED suggested that nocturnal motor activity recorded by actigraphy was reduced and subjective sleep quality was improved. However, there was no change in the frequency and duration of waking episodes related to eating behaviors [6]. In contrast to this paper, we found that pramipexole resulted in complete disappearance of SRED episodes for at least 10 months in our case. To our knowledge, this is the first paper suggesting that pramipexole is effective in a patient with SRED.

 Several other drugs were suggested to be effective against SRED [5, 7]. The antiseizure agent topiramate is possibly the most potent drug. In a retrospective study on 30 consecutive patients with SRED, the data of 25 patients were available; among these 25 subjects, 68% were considered topiramate responders. Adverse effects were reported in 84% subjects [8]; thus, tolerability was a concern for topiramate [8, 15], while pramipexole showed good tolerability [6].

Psychotherapy has been shown to be ineffective for SRED [5]; hence, pharmacotherapy was recommended. However, psychological stress may develop during the course of SRED. In our patient, SRED episodes increased during the psychologically stressed period, and she had a history of depression. Previous reports showed that stressful events were associated with the onset of SRED in approximately 16% of cases [4, 5] and the associated psychiatric diseases were often observed [5]. These observations suggest that psychotherapy is necessary to complement the overall treatment plan, even if psychotherapy alone cannot prevent nocturnal eating.

Another concern of SRED is hypernutrition since SRED patients are often overweight [5]. A slight improvement in plasma HbA1c level was observed after the improvement of SRED episodes. However, SRED was not a key factor of glucose control, and more efforts are necessary to achieve good control of T2DM in our patient.

 The reason of discrepancy in the effects of pramipexole between previous report and our case is not clear. Triazolam apparently worsened the SRED [5], and clonazepam appeared to reduce the effects of triazolam and improve SRED. Thus, there was a possibility that a combination of dopaminergic agent and clonazepam might enhance the effects. A similar phenomenon was previously reported in which carbidopa/l-dopa, codeine, and clonazepam improved SRED and had a higher response rate than monotherapy with dopaminergic agent [5]. Psychotherapy might induce additional effects on those of pharmacotherapy in our patient. Moreover, there is a possibility that our patient is specifically sensitive for the pramipexole treatment. Therefore, further studies with a combination of clonazepam and pramipexole are warranted.

This is the first paper to report that the combination of clonazepam and pramipexole reduced the frequency of SRED episodes and RLS symptoms. Pramipexole had superior tolerability as compared to topiramate, which is reported to be useful in the treatment of SRED. A combination therapy of clonazepam and pramipexole is recommended for patients with SRED associated with RLS as a safe trial. Pramipexole is expected to improve, at least, parasomnia and RLS associated with SRED.
